# Changed expression of placental transporters and disrupted epigenetic patterns in a rat model of schizophrenia

**DOI:** 10.3389/fphar.2025.1673124

**Published:** 2025-11-13

**Authors:** Péter Szatmári, Adrienn Seres-Bokor, Anita Sztojkov-Ivanov, Gabriella Kékesi, Gyöngyi Horváth, Eszter Ducza

**Affiliations:** 1 Department of Pharmacodynamics and Biopharmacy, Faculty of Pharmacy, University of Szeged, Szeged, Hungary; 2 Department of Physiology, Albert Szent-Györgyi Medical School, University of Szeged, Szeged, Hungary

**Keywords:** schizophrenia, placenta, P-glycoprotein, breast cancer resistance protein, fexofenadine, epigenetic, rat

## Abstract

**Introduction:**

Generally, the pregnant women with schizophrenia have higher consumption of medicinal drugs. During pregnancy, placental ABC transporters regulate drug disposition and are involved in fetal and placental development. This study examined the expression and function of placental P-glycoprotein (P-gp) and breast cancer resistance protein (BCRP) transporters *in vivo* and evaluated the epigenetic impact of schizophrenia on the placenta in a rat model.

**Methods:**

The expression of placental P-gp and BCRP was measured by RT-PCR and Western blot techniques in schizophrenia-like Wisket and control Wistar rats on gestation days 15, 18, 20, 21, and 22, while the histone acetyltransferase activity and global methylation state of the placenta were detected by colorimetric kits. Fexofenadine was administered *per os* (10 mg/kg) to pregnant rats and plasma concentrations of fexofenadine were determined with HPLC analysis on the 21 and 22 days of gestation.

**Results:**

Reduced placental P-gp expression was identified in late pregnancy, while the placental BCRP expression upregulation was observed before term in schizophrenia. Significantly lower fetal fexofenadine plasma concentration was measured on the 21st and 22nd days of pregnancy compared to the mother; in contrast, the fexofenadine concentration was similar in the schizophrenia-like mother and fetus. Decreased placental histone acetyltransferase activity and DNA hypermethylation were revealed before term in schizophrenia-like rats.

**Conclusion:**

Based on our results, we can conclude that the expression and function of the placental efflux proteins we examined are altered in schizophrenia, and possibly as a result, altered substrate concentrations were measured in the fetuses. We hypothesize that the altered protein expression may also be a result of the disease-induced epigenetic pattern changes. This study presents novel disease-associated placental ABC transporter alterations, which highlights the dangers of using transporter substrates, especially P-gp, during pregnancy.

## 1 Introduction

Schizophrenia is one of the most severe mental conditions among psychiatric disorders, which affects around 23 million people (0.29%) worldwide (Jauhar et al., 2022; Zhan et al., 2025; World Health Organization, 2025). Although the actual occurrence in the population is probably underestimated, in the last 3 decades, the global prevalence and incidence of schizophrenia have grown by circa 65% and 37%, respectively, an increasing trend for the following years (Solmi et al., 2023). Currently, approximately half of the patients in mental hospitals live with schizophrenia (World Health Organization (WHO) 2025). The onset of schizophrenia generally begins in young adulthood, and women usually have the first episode of psychosis between the ages of 25 and 35 years, which overlaps with the peak of women’s reproductive period (Dazzan, 2021). Pregnant women with schizophrenia also have a high population increase in those decades and the current pregnancy rate has reached the 50%–60% values among women with schizophrenia, which is similar to the general population (Safont et al., 2023).

The exact etiology of schizophrenia is currently unknown. However multiple interactions of several factors like genetic risks, environmental impacts, or neurodevelopmental alterations are presumably responsible for the disease development via epigenetic modulation (Föcking et al., 2019; Smigielski et al., 2020). Nevertheless, the role of brain neurotransmitters’ dysregulations in the pathophysiology of schizophrenia is proven including the imbalance of dopamine, serotonin, glutamate, and gamma-aminobutyric acid (GABA) (Patel et al., 2014; Yang and Tsai, 2017; Stahl, 2018; Luvsannyam et al., 2022). Due to the diverse neurochemical alterations and the interaction of neurotransmitter pathways, schizophrenia is characterized by a wide range of manifestations and classified into positive, negative or cognitive symptoms (Yang and Tsai, 2017). In addition to the various symptoms, mothers with schizophrenia also have an increased hazard to develop pregnancy, delivery, or neonatal complications (Fabre et al., 2021).

Antipsychotic drugs are the main pillars in the treatment of schizophrenia and usually require long-term use of 1–5 years or even decades to control the psychotic episodes and prevent relapses (Tiihonen et al., 2018). More case studies proved that the continuous use of antipsychotics provides sustained remission, while patients relapse if they stop the use of medication during pregnancy (Teodorescu et al., 2017). Based on this, risk-benefit assessments recommend the continuous use of antipsychotics during pregnancy to avoid the recurrence of psychotic episodes (Betcher et al., 2019; Edinoff et al., 2022). Therefore, the rate of medication consumption is significant in pregnant women with schizophrenia, which could further increase in the presence of schizophrenia-related comorbidities. Based on these, placental protective functions are even more important to regulate fetal drug exposure in this special population (Ventriglio et al., 2015; Reutfors et al., 2020).

Placental ATP-binding cassette (ABC) transporters are ATP-dependent transmembrane proteins one of the significant determinants of fetal and placental protection. With the hydrolysis of ATP molecules, they can change conformations and pump out xenobiotics and toxicants out of the placenta back into the maternal blood with efflux pump activity (Szatmári and Ducza, 2023). Efflux pumps are usually identified with medicinal drug transport, however, they also have a relevant role in the regulation of biological processes (Moore et al., 2023). P-glycoprotein (ABCB1, P-gp) and breast cancer resistance protein (ABCG2, BCRP) are the most significant efflux transporters in fetal protection. They are already taken into consideration by the regulatory authorities in the International Council for Harmonization (ICH) M12 guideline, which recommends evaluating whether the novel therapeutics are associated with P-gp or BCRP transport (ICH Harmonised Guideline on Drug Interaction Studies M12, 2022).

Various acute and chronic disease states are associated with P-gp (encoded by the *ABCB1* gene in humans, *Abcb1a* or *mdr1a* and *Abcb1b* or *mdr1b* genes in rodents) and BCRP (encoded by the *ABCG2* gene in humans, *Abcg2* in rodents) transporter alterations in different organs, including placental tissues (Evers et al., 2018; Kozlosky et al., 2022; Moore et al., 2023; Szatmári and Ducza, 2023). Pathologic states are able to impair the epigenetic pattern of the placenta as well, which could be a significant role in the aberrant gene regulation of placental ABC transporters. DNA methylation and histone modifications are the main epigenetic regulators involved in transcriptional activity and chromatin folding, and several *in vitro*, *in vivo* and clinical studies reported that these epigenetic processes are dysregulated in schizophrenia (Kozlosky et al., 2022; Lesch, 2021; Delphin et al., 2024).

In this study, we aimed to determine the expression of placental P-gp and BCRP during pregnancy in schizophrenia-like Wisket model rats and evaluate the efflux pump function via fexofenadine substrate of P-gp *in vivo*. Furthermore, we examined the epigenetic pattern of the placenta at the level of histone acetylation mechanisms and global DNA methylation.

## 2 Materials and methods

All experiments involving animals were complied with the ARRIVE guidelines and carried out in accordance with the Guidance on the operation of the Animals (Scientific Procedures) Act 1986, European Communities Council Directive (2010/63/EU) and the Hungarian Act for the Protection of Animals in Research (Article 32 of Act XXVIII) and with the approval of the National Scientific Ethical Committee on Animal Experimentation (registration number: XIV/1421/2023.). Animals were kept in regulated rooms with 12 h light/dark period, 22 °C ± 3 °C temperature and 30%–70% relative humidity. Standard rodent chow (Animalab Ltd., Vác, Hungary) and bottled tap water were provided *ad libitum*.

### 2.1 Animal model

**FIGURE 1:**
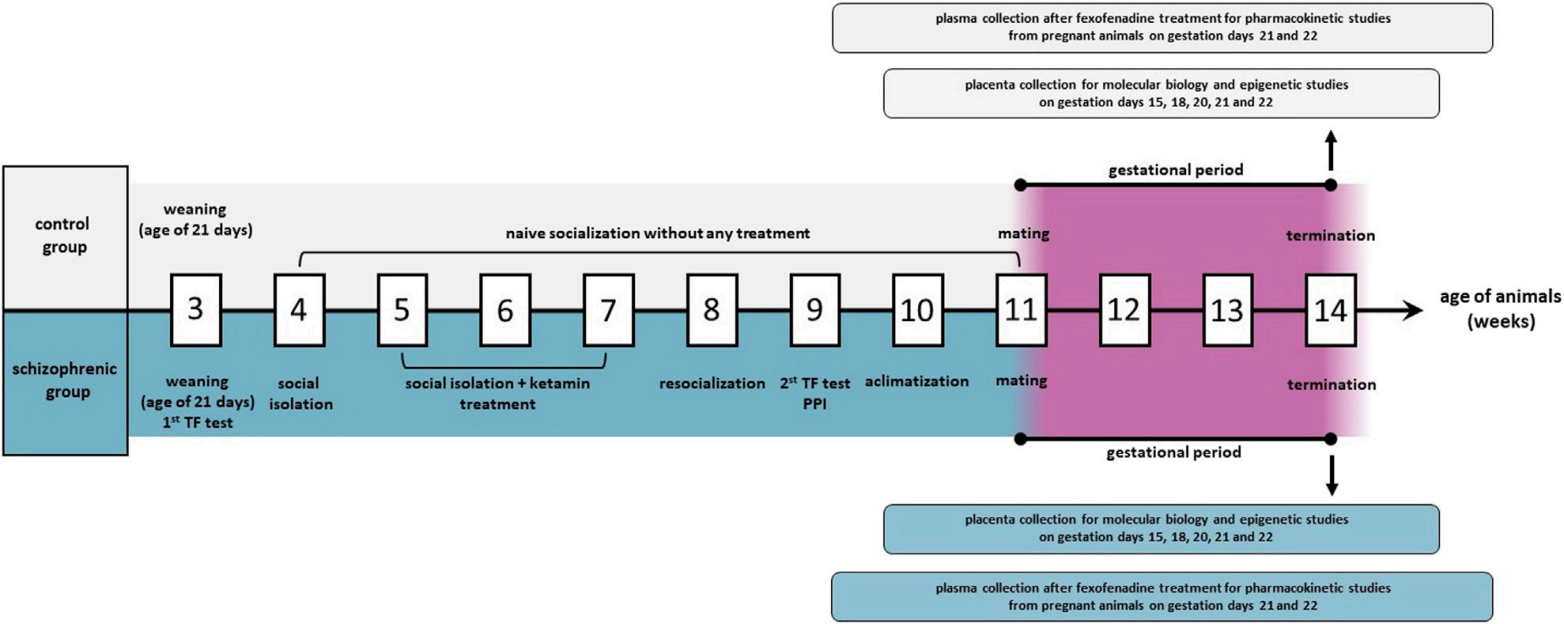
The experimental design of animal studies. TF: tail flick test; PPI: pre-pulse inhibition procedure.

As a model of schizophrenia, Wisket rats were used in our experiments. Wisket rats originate from the Wistar strain by selective breeding through generations according to the rat’s behavioral phenotype, such as pain sensitivity (Tail Flick test, TF), cognitive function, and sensory gating properties (pre-pulse inhibition procedure, PPI), after post-weaning social isolation and sub-chronic ketamine treatment (Figure 1). The development process was maintained as previously described in detail (Petrovszki et al., 2013; Kekesi et al., 2015). Since the animal model was derived from Wistar rats, naive socialized Wistar animals (Toxi-Coop Ltd., Budapest, Hungary) without ketamine treatment were involved in the experiment as the control group (Büki et al., 2019).

### 2.2 Animal experiments

The sexually matured 11 weeks old (200–250 g) schizophrenia-like (n = 42) and control female (n = 42) rats were mated in a special mating box with males (240–260 g) from the same experimental group (n = 6/group) as the females. The previous day of mating between 03:00-04:00 p.m., the proestrus stage of the estrus cycle was determined by a Rat Vaginal Impedance Checker (MK-12, Muromachi Kikai Co., Ltd., Tokyo, Japan). Female animals with 3.0 kΩ vaginal impedance or higher were chosen for the mating process for the following morning. The selected female and male rats were separated in the mating box by an electronic metal door, which was opened automatically between 03:00-05:00 a.m. and provided the opportunity of copulation until 08:00-09:00 a.m. Vaginal smear samples were taken and checked with microscope at ×20 magnification. Visible copulation plug in the vagina or detected sperm in the vaginal smear confirmed the presence of pregnancy and regarded the animal as a first-day-old pregnant dam. Pregnant animals were selected for molecular biology or pharmacokinetic studies and both experimental groups were divided into subgroups (n = 6/subgroup) representing the different gestational days. Male animals were only used for mating.

For molecular biology studies, pregnant rats on gestation days 15, 18, 20, 21 and 22 were terminated under deep isoflurane anesthesia by exsanguination and placental tissues were randomly removed (6-8/dam) and placed into RNAlater Solution (Sigma Aldrich, Budapest, Hungary).

Pregnant animals involved in pharmacokinetic investigations were fasted for 16 h before the treatment, and on gestation days 21 and 22 (n = 6/day), they were administered *per os* with a single dose of 10 mg/kg fexofenadine (Fex). The substrate, dose and cupping time were determined by the literature data (Qiang et al., 2009; Jaisue et al., 2010; Pinto et al., 2021; ICH Harmonised Guideline on Drug Interaction Studies M12, 2022; Popova et al., 2023). Fexofenadine tablet (Allegra 120 mg Tablet, Opella Healthcare Commercial Ltd., Budapest, Hungary) was grinded and suspended in 0.25% mucilage methylcellulose (Sigma Aldrich, Budapest, Hungary). Maternal and fetal blood were collected into tubes containing Na_2_EDTA after 1 h of administration. All fetuses were surgically removed from the mothers under isoflurane anaesthesia, then separated from the uterus, placenta, amniotic membrane and umbilical cord. Pups were washed from maternal blood and amniotic fluid by sterile isotonic water (NaCl Kabi 9 mg/mL Injection Solution, Fresenius Kabi Hungary Ltd., Budapest, Hungary) to avoid the contamination. The purified fetuses were decapitated and fetal blood samples were collected. Fetal sex has not been determined due to technical limitations; therefore, sex-specific effects were not evaluated. Blood samples of mothers were taken by cardiac puncture. All blood samples were centrifuged with SIGMA 1–15 K Centrifuge (Sigma Laborzentrifugen GmbH, Germany) at 4000 g, 10 min and 4 °C to separate plasma. The collected placental tissues and plasma aliquots were frozen at −80 °C until molecular biology and bioanalytical measurements. Our experimental design is graphically represented in Figure 1.

### 2.3 Molecular biology studies

#### 2.3.1 Total RNA, DNA and protein isolation from placental tissues

Placental tissues were mechanically smashed by grinding balls with a Sartorius MikroDismembrator U (Sartorius, Göttingen, Germany) ball mill. Total cellular RNA was extracted from the homogenized samples by guanidinium thiocyanate-acid-phenol-chloroform according to the procedure described previously (Chomzynski, 1987). The total cellular DNA was isolated by Geneaid™ DNA Isolation Kit (Central European Biosystem Ltd., Hungary) from the powder of placental tissues following the instruction manual.

For protein isolation, powdered samples were homogenized with a solution of RIPA lysis, extraction buffer and protease inhibitor cocktail. The supernatant layer was then used to measure total protein. The concentrations of RNA, DNA, and protein samples were measured with BioSpec Nano (Shimadzu, Japan) spectrophotometer.

#### 2.3.2 RT-PCR

1 µg of total RNA and TaqMan RNA-to-C_T_-Step One Kit (Thermo Fisher Scientific, Hungary) were used for reverse transcription and amplification performed by ABI StepOne Real-Time cycler as described previously (Büki et al., 2019). The following primers were used: assay ID Rn01639253_m1 for the *Abcb1a*, Rn01529252_g1 for the *Abcb1b,* Rn00710585_m1 for the *Abcg2*, and Rn00667869_m1 for *β-actin* as endogenous control (ThermoFisher Scientific, Hungary).

#### 2.3.3 Western blot analysis

50 μg of protein per well was electrophoresed and blotted on nitrocellulose membranes then antibody binding was detected with the WesternBreeze Chromogenic Western blot immunodetection kit (ThermoFisher Scientific, Hungary) as noted before (Büki et al., 2019). The blots were incubated with the following primary antibodies: MDR1 (141 kDa, 1:300, bs-0563R, Bioss Antibody), ABCG2 (72 kDa, 1:500, SAB5701106, Merck Life Science Ltd., Hungary) and β-actin (42 kDa, 1:1000, bs-0061R, Bioss Antibody).

#### 2.3.4 Colorimetric assays

According to the manufacturers' manual the placental relative methylation state was measured with Imprint® Methylated DNA Quantification Kit (Sigma-Aldrich, Hungary) and histone acetyltransferase activity was determined by HAT Activity Colorimetric Assay Kit (Sigma-Aldrich, Hungary).

### 2.4 Pharmacokinetic studies

#### 2.4.1 Chemical and reagents

Fexofenadine hydrochloride (pharmaceutical primary standard and grade), cetirizine hydrochloride (pharmaceutical secondary standard grade), analytical grade potassium dihydrogen phosphate (KH_2_PO_4_) and analytical grade glacial acetic acid were purchased from Merck (Darmstadt, Germany). Gradient grade acetonitrile was obtained from HiPerSolv Chromanorm (VWR International Kft., Budapest, Hungary).

#### 2.4.2 Plasma sample preparation for HPLC analysis

200 µL plasma sample was spiked with 20 µL of cetirizine internal standard (Bharathi et al., 2008; Flynn et al., 2011; Yao and Srinivas, 2012) working solution (50 μg/mL) and 100 µL 5% acetic acid solution to acidify the sample. Proteins were precipitated with 1.5 mL acetonitrile. The mixture was vortexed for 1.5 min and centrifuged at 12,000 rpm for 10 min at 4 °C. The supernatant was transferred to another clean Eppendorf tube and evaporated to dryness under a stream of nitrogen at 40 °C. The residue was reconstituted in 100 µL mobile phase and vortex-mixed for 10 s, and 20 µL solution was injected for HPLC analysis.

#### 2.4.3 HPLC chromatographic conditions

Fexofenadine quantification in plasma was performed using high-performance liquid chromatographic (HPLC) method. The Shimadzu HPLC system (Simkon Ltd., Budapest, Hungary) was equipped with an LC-20AD solvent delivery system, a DGU-20A3 online degasser, an SIL 20A HT autoinjector, a CTO-20A column oven, an SPD-M20A photodiode-array detector and a CBM-20A system controller. The system control and data acquisition were performed by Shimadzu LC solution 5.106 software (Simkon Kft., Budapest, Hungary). The separations were achieved by reversed-phase liquid chromatography on a Phenomenex Kinetex C8 100A (4.60 mm × 150 mm, 5 µm) analytical column (Gen-Lab Ltd., Budapest, Hungary), protected by a guard column. The column temperature was kept constant at 30 °C. The mobile phase consisted of 0.01 M KH_2_PO_4_ buffer (pH 3.3) and acetonitrile (40:60, v/v) pumped at a flow rate of 1 mL/min. The mobile phase was filtered by a Millipore vacuum filtration system (Merck, Darmstadt, Germany) equipped with a 0.45 µm pore size filter and degassed by ultrasonication. The detection wavelength was 230 nm.

A stock solution of fexofenadine was prepared in acetonitrile at a concentration of 1 mg/mL. A stock solution of the cetirizine internal standard was prepared in acetonitrile at a concentration of 1 mg/mL. The working solutions of fexofenadine were prepared by diluting the stock solution with acetonitrile, and then working solutions were obtained by spiking 980 µL of drug-free rat plasma with 20 µL of the appropriate fexofenadine stock solution, resulting in calibration standards at the following concentrations: 0.02, 0.05, 0.10, 0.20, 0.50, 1.00, 2.00, 5.00 and 10.00 μg/mL. All of the solutions were stored at −20 °C.

The HPLC–UV method was partially validated according to the Bioanalytical Method Validation guidance by the US FDA (Food and Drug Administration Center for Drug Evaluation and Research Center for Veterinary Medicine, 2018). The validation parameters included linearity, lower limit of quantification, selectivity, sensitivity, accuracy, precision, and recovery.

Selectivity of the method was examined by analyzing blank rat plasma samples from six individual rats, comparing the chromatograms of blank plasma spiked with fexofenadine and IS. No interfering components are observed at the retention times of fexofenadine or the IS in the blank samples (Supplementary Figure S1).

Calibration standards (0.02, 0.05, 0.10, 0.20, 0.50, 1.00, 2.00, 5.00, and 10.00 μg/mL) were prepared by spiking blank rat plasma with working standard solutions of known concentrations of fexofenadine and cetirizine. The calibration curve was measured in triplicate over two different days and regressed using the ratio of peak areas of fexofenadine/cetirizine versus the nominal concentration. The linearity of the method was evaluated using least-squares linear regression analysis without a weighted factor. The average equation of the calibration curve was Y = 0.04322X – 0.004053 (*R* ^2^ = 0.9978 ± 0.0008), demonstrating the linearity of the method. The accuracy of the back-calculated concentrations was calculated as accuracy (%) = (measured value/nominal value) × 100, while precision was expressed as the percentage of coefficient of variation, %CV = (standard deviation/mean) x 100. The accuracy and precision of back-calculated concentrations were within the ±15% acceptance criteria. Supplementary Figure S2 presents the mean calibration curve for fexofenadine in rat plasma, and Supplementary Table S1 summarizes the calibration curve results. The sensitivity of the HPLC method was characterized by the lower limit of quantification (LLOQ). The LLOQ of fexofenadine in rat plasma was 0.02 μg/mL, with an accuracy of 101.73% and a precision of <7.02%; both values being lower than the ±20% acceptance criteria, thus the method can be considered sensitive (Supplementary Table S2).

QC samples in three concentration levels (n = 5) were measured on two different days to obtain the intra- and interday accuracy and precision. The intra-day accuracy and precision of QC samples were in the range of 85.12%–106.41% and 6.68%–9.76%, respectively. The inter-day accuracy and precision of QC samples ranged from 85.50% to 102.99% and from 7.45% to 9.36%, respectively. The results are summarized in Supplementary Table S2.

The extraction recovery was detemined by calculating the percentage of peak area ratio of fexofenadine in pre-spiked and post-spiked QC samples in three concentration levels (n = 6). The recovery of fexofenadine ranged from 95.9% to 106.1% in rat plasma (Supplementary Table S3).

### 2.5 Statistical analysis

All data were analyzed by Prism 9.0 software (Graphpad Software Inc. San Diego, CA, USA). The normal distribution of the sample data was verified using the Shapiro-Wilk test. In molecular biology and epigenetic studies, two-way ANOVA was used to examine the effect of the experimental intervention and gestation period. One-way ANOVA was used to evaluate the effect of the experimental intervention on the maternal and fetal plasma concentrations of fexofenadine. Bonferroni *post hoc* test was applied for both statistical types. Unpaired t-test was used to compare the number of the fetuses between the two experimental group. Each is presented as the mean ± standard error of the mean (SEM). Significance was accepted at p < 0.05.

## 3 Results

### 3.1 Molecular biology studies on the expression of placental P-gp and BCRP

**FIGURE 2:**
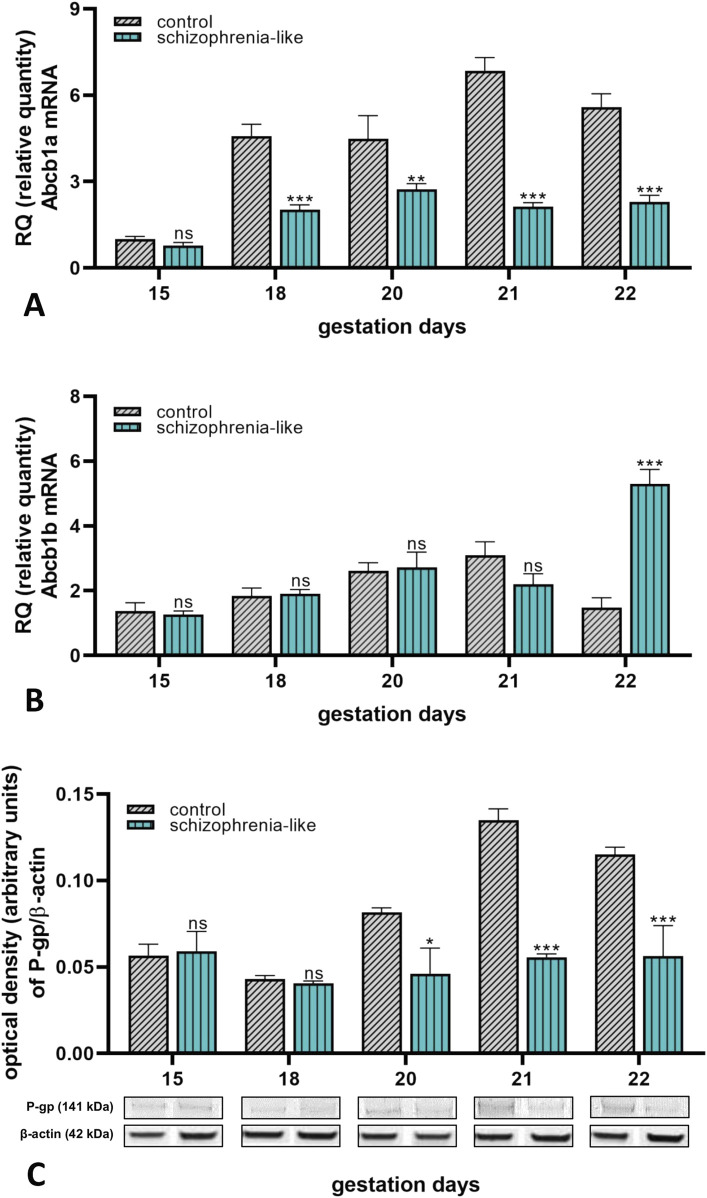
Changes of placental Abcb1a **(A)** and Abcb1b **(B)** mRNA and P-gp **(C)** protein expressions in control and schizophrenia-like pregnant rats on different gestational days (15, 18, 20, 21, 22). Data are presented as means ± SEM and statistical significance was accepted at p < 0.05 compared to the control group. (ns p > 0.05, *p < 0.05, **p < 0.01, ***p < 0.001). n = 6/gestational day in each experimental group. The original gel images of the Western blot measurements are attached in the Supplementary Material (Supplementary Figure S3).

To evaluate the effect of schizophrenia on the gene expression of placental ABC transporters, mRNA and protein expressions of the selected transporters were analyzed during late pregnancy. Molecular biology studies of the placenta revealed a significant reduction of *Abcb1a* mRNA expression from gestation day 18 until 22 in the schizoid animals than the control group (Figure 2A). Expression of *Abcb1b* remained unchanged but tripled on the last day of gestation in schizophrenia-like pregnant rats compared to controls (Figure 2B). The P-gp protein expression correlated with the *Abcb1a* mRNA expression and showed reduction on pregnancy days 20, 21 and 22 (Figure 2C).

**FIGURE 3:**
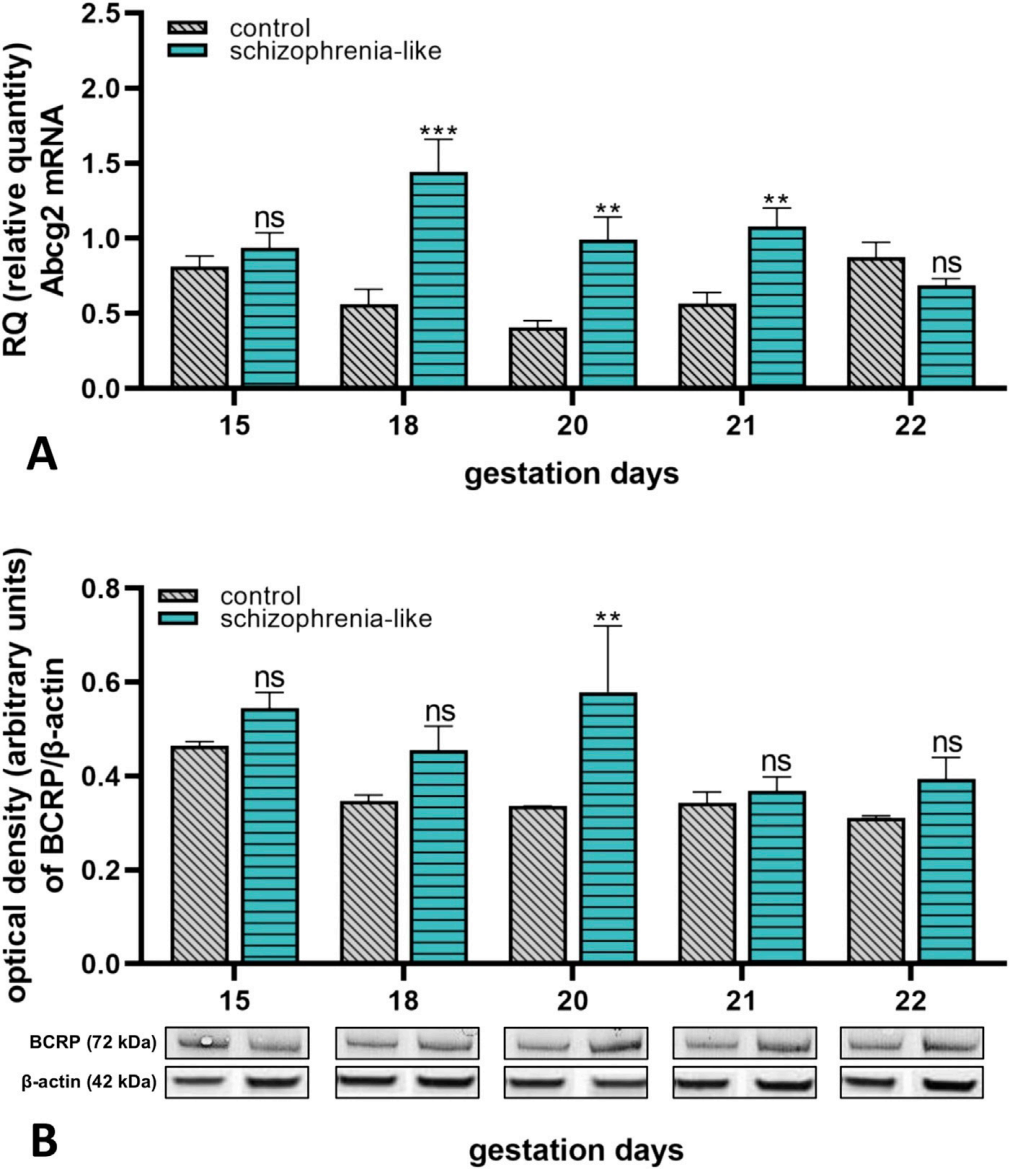
Changes of placental Abcg2 mRNA **(A)** and BCRP **(B)** protein expressions in control and schizophrenia-like pregnant rats on different gestational days (15, 18, 20, 21, 22). Data are presented as means ± SEM and statistical significance was accepted at p < 0.05 compared to the control group (ns p > 0.05, **p < 0.01, ***p < 0.001). n = 6/gestational day in each experimental group. The original gel images of the Western blot measurements are attached in the Supplementary Material (Supplementary Figure S3).

The *Abcg2* mRNA expression of placenta from schizophrenia-like rats was significantly increased on gestation day 18, 20 and 21 compared to the control groups (Figure 3A). The BCRP protein expression was increased on all gestation days; however, it was significant only on gestation day 20 in rats with schizophrenia phenotype (Figure 3B).

### 3.2 Pharmacokinetic studies on fetal fexofenadine exposure

**FIGURE 4:**
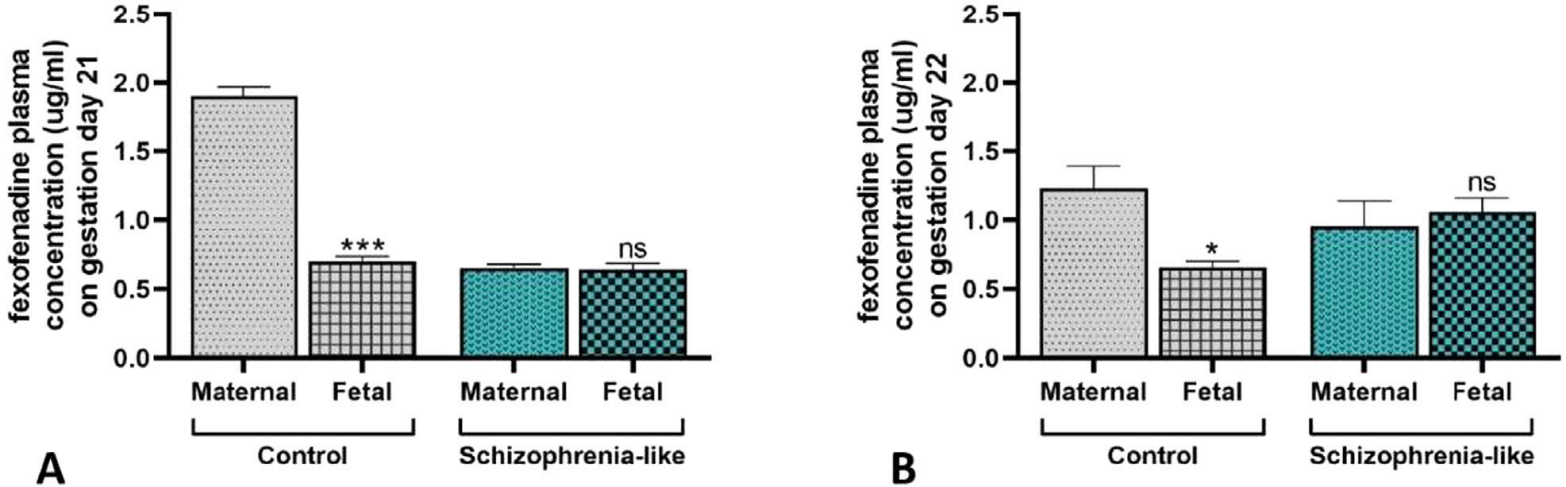
Fexofenadine concentration in fetal and maternal plasma in control and schizophrenia-like pregnant rats on gestation days 21 **(A)** and 22 **(B)**. Data are presented as means ± SEM and statistical significance was accepted at p < 0.05 (ns p > 0.05, *p < 0.05, ***p < 0.001 compared to the maternal plasma). n = 6 dam in each experimental group treated with fexofenadine. Representative chromatograms of experimental plasma samples are attached in the Supplementary Material (Supplementary Figure S4).

To assess the impact of the schizophrenia-associated reduced placental P-gp expression on the fetal substrate concentration, maternal and fetal Fex plasma concentration were measured following *per os* administration. The Fex plasma concentration was significantly lower in the fetuses on gestation days 21 (Figure 4A) and 22 (Figure 4B) compared to their mothers in the control groups, with the difference of approximately 1.20 and 0.75 μg/mL, respectively. However, in schizophrenia-like animals, there were no significant differences between the mothers and the fetuses in the Fex concentrations on gestation days 21 (Figure 4A) and 22 (Figure 4B). The number of fetuses did not change in the schizophrenic group (11.47 ± 0.375) compared to controls (11.91 ± 0.791; p > 0.05).

### 3.3 Epigenetic studies on HAT activity and DNA methylation level

**FIGURE 5:**
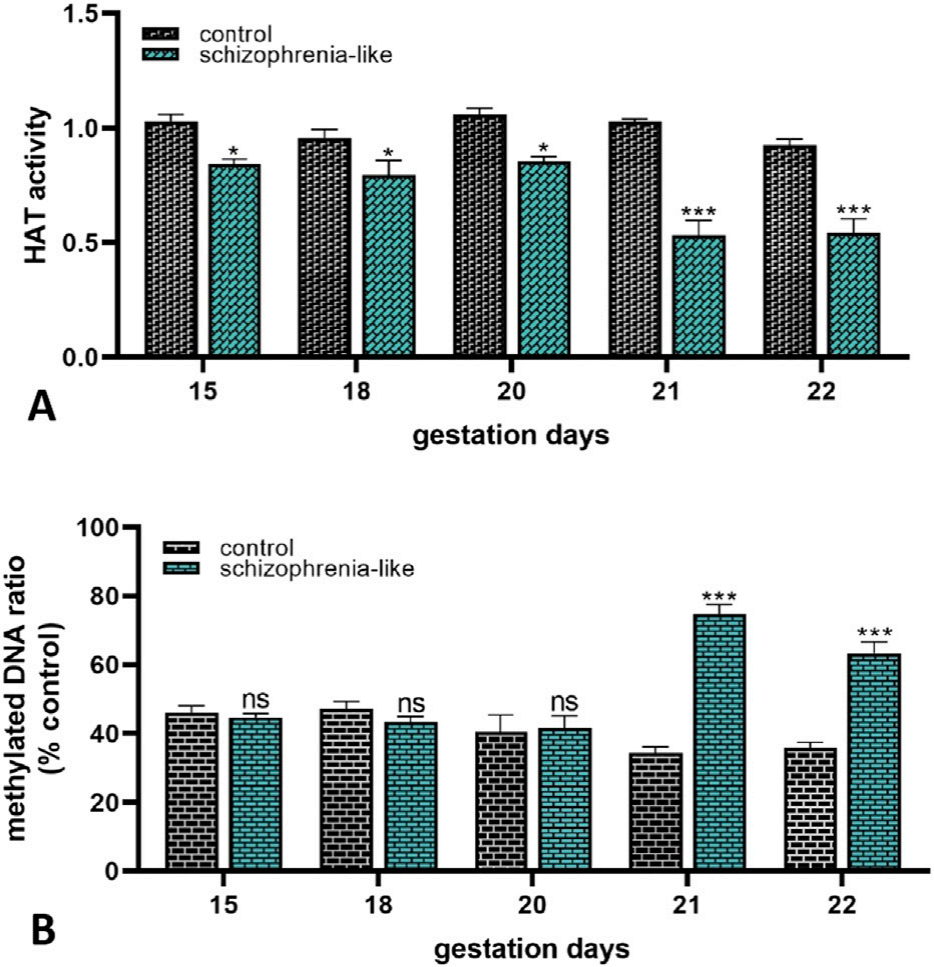
Epigenetic changes of placenta on histone-acetylation **(A)** and DNA methylation **(B)** in control and schizophrenia-like pregnant rats on different gestational days (15, 18, 20, 21, 22). Data are presented as means ± SEM and statistical significance was accepted at p < 0.05 compared to the control group (ns p > 0.05, *p < 0.05, ***p < 0.001). n = 6/gestational day in each experimental group.

Finally, to establish whether schizophrenia altered the epigenetic profile of the placenta in late pregnancy, global epigenetic mechanisms were investigated. In schizophrenia-like rats, the placental histone acetyltransferase activity was significantly lower from gestation day 15–22 than the controls (Figure 5A). The methylated DNA levels of the placenta did not change until gestation day 20. In contrast, significantly higher methylated status was revealed on the last 2 days of gestation compared to the control groups (Figure 5B).

## 4 Discussion

Although several disease-associated transporter alterations have been identified (Kozlosky et al., 2022); however, no data are available in any species about the expression of placental ABC transporters in schizophrenia. Therefore, the aim of this recent study was to investigate this interesting and important change in transporter expression. To our knowledge, this was the first time to examine the effect of pre-existing schizophrenia on the expression and function of placental P-gp and BCRP transporters in a rat model and evaluate the efflux pump function of P-gp *in vivo* with orally administered fexofenadine substrate.

The gestational-age-dependent placental P-gp (Novotna et al., 2004) and BCRP (Yasuda et al., 2005; Cygalova et al., 2008) expression alterations in Wistar rats were previously described and we got similar expression patterns in our control animals. We determined reduced *Abcb1a* mRNA expression which was similar as P-gp expression after the 18th day of gestation until term and increased *Abcb1b* level on pregnancy day 22 in schizophrenia-like rats. The *Abcb1a* and *Abcb1b* isoforms have partly different expression regulation (Szatmári et al., 2025), which explains their different expression levels within the tissue. In rodents, *Abcb1a* pre-dominance is identified as related to the placenta (Schinkel et al., 1997; Lankas et al., 1998a; Fujita et al., 2022), as also seen in our animals from gestation day 20. The difference in protein expression from the *Abcb1a* isoform pattern before gestation day 20 proves that the two genes together form the final P-gp expression. In rats with schizophrenia, placental *Abcg2* mRNA expression increased from gestation day 18–21, while BCRP expression increased on all gestation days, but it was only significant on gestation day 20, which is probably caused by the varied post-transcriptional or post-translational processes.

More studies with *Abcb1a/1b* and *Abcg2* knock-out pregnant mice models evaluated the role of placental P-gp and BCRP transporters in fetal drug distribution and revealed higher fetal substrate exposure and substrate-specific fetal abnormalities compared to the wild-type (Lankas et al., 1998b; Smit et al., 1999; Zhang et al., 2007; Nishimura et al., 2024). Studies also exist investigating P-gp and BCRP efflux function in healthy and pathological pregnancies in rodents with substrates like lopinavir (Anger and Piquette-Miller, 2011), digoxin (Wang et al., 2015), fexofenadine (Popova et al., 2023), nitrofurantoin (Zhang et al., 2007), glyburide (Zhou et al., 2008), cimetidine (Cygalova et al., 2008), rosuvastatin (Dai and Piquette-Miller, 2024) or tadalafil (Nishimura et al., 2024). In these studies, an inverse correlation is identified between efflux pump expression in the placenta and their substrates concentrations in the fetus (Moore et al., 2023). Fexofenadine is a well-identified substrate, which is used as a probe drug to evaluate placental P-gp function in *ex vivo* and *in vivo* studies (Qiang et al., 2009; Pinto et al., 2021; Popova et al., 2023). Fexofenadine is also a practical option in clinical studies because it is safer and possesses lower risks than the widely used digoxin with a narrow therapeutic window (Gaspar et al., 2025). The ICH M12 guideline with the participation of the Food and Drug Administration and European Medicines Agency also recommends its use as a substrate (ICH Harmonised Guideline on Drug Interaction Studies M12, 2022; FDA Drug Development and Drug Interactions, 2025). Furthermore, fexofenadine is frequently used to treat allergic conditions during pregnancy (Andersson et al., 2020).

In our study, animals were treated *per os*, because oral administration is the usual way to take medicine, and the impact of the absorption process on fetal substrate exposure could be evaluated. We did not observe any difference between maternal and fetal fexofenadine plasma concentrations in the schizophrenia-like animals compared to controls. It can be assumed that the reduced P-gp expression in the schizophrenia-like animals results in an insufficient barrier function in the placenta, which was likely unable to limit the exposure of fexofenadine to the fetus and leads to similar plasma concentrations in both the fetal and maternal compartments, in contrast to the controls. Furthermore, the maternal plasma concentration of fexofenadine was reduced in schizoid animals, suggesting that schizophrenia may also affect the absorption of fexofenadine.

It is important to mention that *in vivo* functional studies of P-gp are complicated by the fact that most of the substrates used as a probe are not specific for P-gp. Fexofenadine is also a known substrate of organic anion transporting polypeptide (OATP) transporter 1B1, 1B3 and 2B1, respectively (ICH Harmonised Guideline on Drug Interaction Studies M12, 2022; FDA Drug Development and Drug Interactions, 2025). These transporters are mainly expressed in the liver and intestine, however OATP2B1 is expressed in the basal membrane of the human placenta while OATP1B1 has been detected in human placental tissues in very low levels (Dallmann et al., 2019; Chen et al., 2020; Ganguly et al., 2021; Yamashita and Markert, 2021). In rats, Oatp1b2 (also known as Oatp4 and the rodent orthologue of human OATP1B1 and 1B3) has minimal expression in the placenta while Oatp2b1 (also known as Oatp9 and the rodent orthologue of human OATP2B1) is expressed in the rat placenta, but much less compared to the liver in contrast to *mdr1a* and *mdr1b* which reach abundant expression in the rat placenta compared to the liver (Leazer and Curtis, 2003; St-Pierre et al., 2004; Liu, 2019). These OATP transporters are less relevant in the rat placenta than P-gp according to the fetal protection and xenobiotic exposure (Leazer and Curtis, 2003) which indicates the dominant role of P-gp in the determination of fexofenadine disposition across the rat placenta.

User rates of P-gp and BCRP substrates are near 10% and 3% among pregnant women (Daud et al., 2015; 2017). A register-based study revealed that the use of P-gp and/or BCRP substrates or inhibitors simultaneously during pregnancy increases the risk of congenital anomalies (Ellfolk et al., 2020). In addition, reduction of placental P-gp and BCRP expressions is associated with fetal death (Nardi et al., 2025). Since placental P-gp is decreased in schizophrenia during pregnancy, it can be assumed that drugs with P-gp transport used in pregnant women with schizophrenia could reach higher concentrations in the feto-placental unit and be harmful for the fetus or placenta contributing to the appearance of drug-induced fetal complications during the pharmacotherapy. To prevent this, the use of P-gp substrate drugs in schizophrenia should be reconsidered. Since BCRP did not indicate higher fetal exposure of BCRP substrates based on its expression changes, pharmacokinetic studies with a BCRP substrate were not performed.

Altered epigenetic modifications in the placenta are linked with several pregnancy complications and ABC transporter dysregulations (Kozlosky et al., 2022; Talpur et al., 2024). The link between epigenetic regulation and the pathophysiology of schizophrenia is also proven (Yang et al., 2025). Since the disruption of multiple regulatory pathways is responsible for the development of varied pregnancy complications and the dysregulation of ABC transporters, global epigenetic profiles of the placenta were investigated. Transcriptional activity could be affected by both and evidence of their reciprocal relationships is described. Histone acetylation is a post-translational modification, when an acetyl-group is added to a histone protein and relaxes the interaction between the tightly rolled DNA chain and the histone protein, leading to an open chromatin structure which is linked with the activation of transcriptional processes. This process is reversible and mediated by the balance of histone acetyltransferases (HATs) and histone deacetylases (HDACs) activities (Lee et al., 2021). DNA methylation alters gene expression without changing the DNA sequence by the reaction of covalent fusion of methyl group and a cytosine nucleotide base (called CpG islands) of DNA. Hypermethylation in the promoter region of a gene is generally associated with a decrease in transcriptional activity and plays a major role in gene silencing, which is essential in cell differentiation and development. Moreover, studies revealed that histone acetylation is associated with DNA demethylation (El-Osta and Wolffe, 2001; Staud and Ceckova, 2015; Gujral et al., 2020; Lee et al., 2020). We observed decreased HAT activity in the schizophrenia-like animals until term while, DNA methylation increased on the last 2 days of pregnancy. Previous studies proved that global HAT activity is positively correlated with the global acetylation changes (Wang et al., 2009; Selvi et al., 2010), which leads us to conclude that the level of global histone acetylation may also decrease with the reduction of HAT activity. Based on this, we believe that due to the presumed histone hypoacetylation, the DNA chain is not able to drift away from the histone protein, resulting in a compact chromatin structure. Since DNA chains permanently remain closed on the histone protein, after a while DNA begins to methylate which contributes to altered transcriptional activity and long-term gene silencing. Numerous studies reported epigenetic impairments in brain, blood, and saliva tissues from patients with schizophrenia (Delphin et al., 2024); however, similar research of placental tissues from mothers with schizophrenia is not available yet. Various pregnancy conditions and complications present altered DNA methylation level in the placenta, including obesity, maternal diabetes (Reichetzeder et al., 2016; Lizárraga et al., 2024; Hjort et al., 2022), preeclampsia (Cruz et al., 2020), caesarean section (Słabuszewska-Jóźwiak et al., 2020), down syndrome (Jin et al., 2013) or large for gestational age (Dwi Putra et al., 2020), and several of these conditions have been linked with placental ABC transporter expression alterations (Kozlosky et al., 2022). A relatively recent study revealed that prenatal stress condition induces hypermethylation on placental ABCB1 CpG sites and regions which is coupled with reduced *Abcb1a/1b* mRNA and protein expressions (Liu et al., 2025). Another study observed lower total and CpG site-specific methylation levels of placental ABCB1 and unaltered methylation levels of ABCG2 in neonatal opioid withdrawal syndrome (Townsel et al., 2025). Enormous evidence is also available that the methylation of ABCB1 promoter is inversely correlated with the mRNA and protein expression of the ABCB1 in prostate cancer and leukemia, while the association of the ABCG2 promoter methylation state and its mRNA and protein expression level is extensively varied by cancer types (Zappe and Cichna-Markl, 2020). Based on this, we presume that impaired epigenetic pattern in schizophrenia may play a role in the dysregulation of placental P-gp during pregnancy. Nevertheless, it is important to mention, that global histone acetylation is a dynamic process regulated by both histone acetylation and histone deacetylation (Wang and Luo, 2025), therefore future studies are needed to determine the exact histone-acetylation levels and perform gene-specific methylation and histone-acetylation analyses to evaluate the direct relationship between the altered placental epigenetic pattern and ABC transporter expression changes in schizophrenia. Although the gene expression activity depends on the exact site of epigenetic modifications on histone protein and the DNA chain, the observed placental epigenetic alterations indicate that epigenetic changes occur as a result of the disease. As the presence of schizophrenia is an increased risk factor for developing pregnancy, delivery, or neonatal complications, the observed schizophrenia-related epigenetic impairments might be associated with these adverse pregnancy outcomes or even disease susceptibility of offspring in later life (Fabre et al., 2021; Talpur et al., 2024).

## 5 Conclusion

In summary, we revealed insufficient barrier function of the placenta against fexofenadine in the schizophrenia-like rat model, which could be primarily caused by the reduced expression of placental P-gp. Since drug utilization in schizophrenia during pregnancy is significant, the use of drugs associated with P-gp transport should be reconsidered. In contrast, based on the expression changes, increased risk is not assumed for BCRP substrate drugs. Altered epigenetic processes in the placenta were also observed in dams with schizophrenia, suggesting their connection with schizophrenia-associated maternal and fetal complications. Our findings may provide a new aspect for drug choice and dose setting during pregnancy under the condition of schizophrenia; however future human investigations are required for the clinical translation of the obtained results.

## 6 Limitations

Our findings face some limitations, which should be considered. Most importantly, the study is lacking in human investigations, which is necessary for the clinical implications for pregnant women with schizophrenia. Another weakness is that only one time-point was investigated after a single dose of fexofenadine treatment, which does not allow the assessment of long-term fetal outcomes. For further evaluation, detailed comparative pharmacokinetic investigations would be needed with the comparison of per os and intravenous administration using more time-points and specific teratogenic substrates and inhibitors. In addition, our results do not provide data about the sex-specific and site-specific placental expression changes of the investigated transporters.

## Data Availability

The raw data supporting the conclusions of this article will be made available by the authors, without undue reservation.
