# Supplementary material for: Changed expression of placental transporters and disrupted epigenetic patterns in a rat model of schizophrenia
Source: Front Pharmacol. 2025 Nov 13;16:1673124. doi: 10.3389/fphar.2025.1673124 (PMC12657358; doi:10.3389/fphar.2025.1673124)
Supplement: Supplementary file 1 [file DataSheet1.pdf]

Supplementary Material

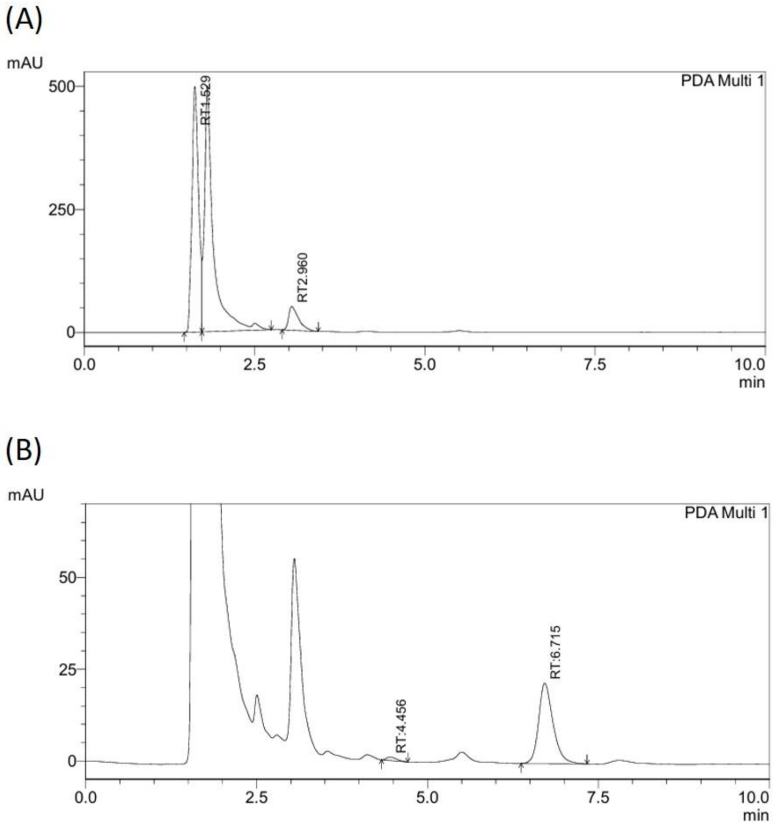

Supplementary Figure S1. Representative chromatograms demonstrating the selectivity of the HPLC–UV method: blank rat plasma (**A**) and plasma spiked with fexofenadine at the low quality control level (0.05 µg/mL) and cetirizine as the internal standard (**B**), respectively.

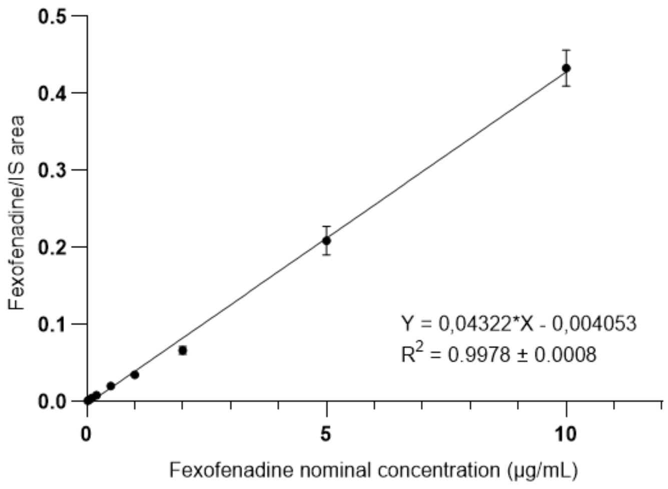

Supplementary Figure S2. Calibration curve of fexofenadine in rat plasma.

Supplementary Table S1. Precision and accuracy data for fexofenadine calibration standards from spiked rat plasma.

Nominal concentration (μg/mL)	Measured concentration ± SD (μg/mL) (n = 6)	Accuracy (%)	Precision (%CV)
0.02	0.020 ± 0.001	101.73	7.02
0.05	0.052 ± 0.004	103.21	8.43
0.1	0.103 ± 0.08	103.32	8.16
0.2	0.199 ± 0.18	99.46	9.19
0.5	0.492 ± 0.016	98.47	3.31
1	0.867 ± 0.087	86.75	10.07
2	1.622 ± 0.069	85.12	4.23
5	4.845 ± 0.249	96.90	5.14
10	10.167 ± 0.712	101.67	7.00

SD, standard deviation; accuracy (%) = (measured value/nominal value) × 100; %CV = (standard deviation/mean) x 100.

Supplementary Table S2. The intra- and interday accuracy and precision data of three QC samples and LLOQ samples of fexofenadine in rat plasma (n = 5).

Nominal concentration (μg/mL)	Intra-day				Inter-day	
	Day 1		Day 2			
	Accuracy (%)	Precision (%CV)	Accuracy (%)	Precision (%CV)	Accuracy (%)	Precision (%CV)
0.02	98.08	8.66	105.78	9.25	101.93	9.36
0.05	106.41	6.68	99.57	7.97	102.99	7.73
2	85.88	7.85	85.12	7.86	85.50	7.45
8	102.01	9.76	97.45	9.06	99.73	9.22

Accuracy (%) = (measured value/nominal value) × 100; %CV = (standard deviation/mean) x 100.

Supplementary Table S3. Recovery of fexofenadine and in rat plasma (n = 6).

Nominal concentration (μg/mL)	Recovery (%)	%CV
0.05	73.34	4.85
2	76.23	7.33
8	77.02	10.60

%CV = (standard deviation/mean) x 100.

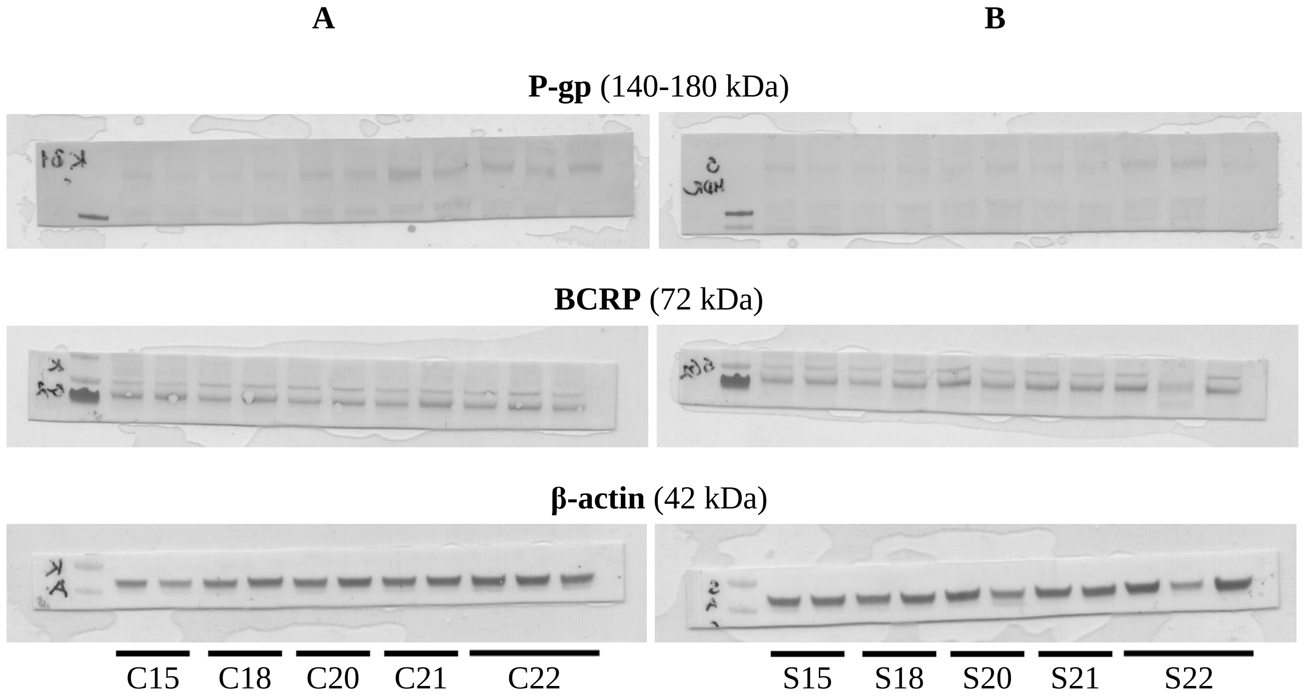

Supplementary Figure S3. Changes of placental P-glycoprotein (P-gp) and breast cancer resistance protein (BCRP) expression in control (Wistar) (**A**) and schizophrenia-like (Wisket) (**B**) rats on different gestation days. Gels were run in paralel with the same conditions. Abbreviations: C, control animals; S, schizophrenia-like animals

(A)

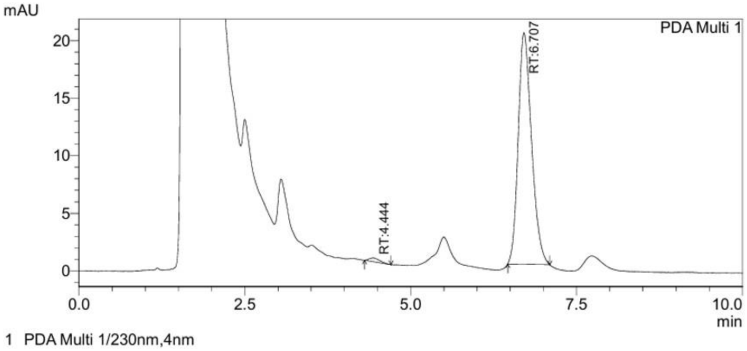

Quantitative Results

PDA

ID#	Name	Ret. Time	Area	Height
1	RT:4.444	4.444	3603	339
2	RT:6.707	6.707	281722	20123

(B)

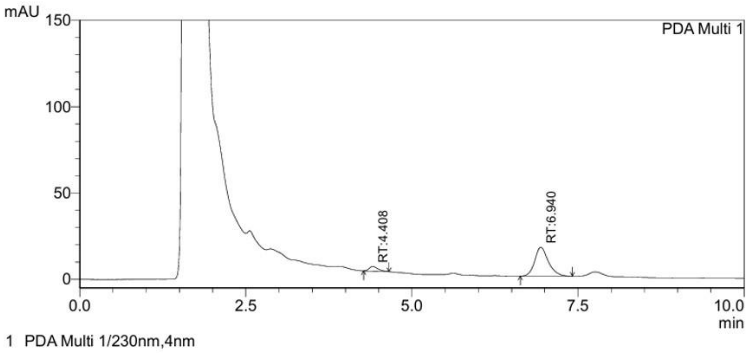

Quantitative Results

PDA

ID#	Name	Ret. Time	Area	Height
1	RT:4.408	4.408	24874	2747
2	RT:6.940	6.940	238042	16718

(C)

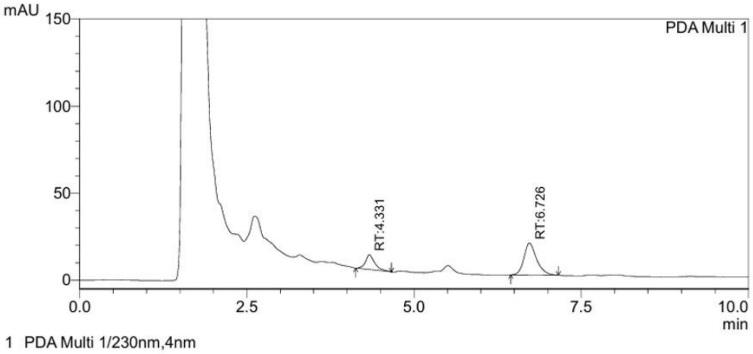

Quantitative Results

PDA

ID#	Name	Ret. Time	Area	Height
1	RT:4.331	4.331	82386	8481
2	RT:6.726	6.726	242053	18292

Supplementary Figure S4. Representative chromatograms of experimental plasma samples exhibiting low (**A**), medium (**B**), and high (**C**) fexofenadine concentrations, respectively, with cetirizine as the internal standard.
